# A multiscale model of the effect of Ir thickness on the static and dynamic properties of Fe/Ir/Fe films

**DOI:** 10.1038/s41598-018-21934-5

**Published:** 2018-03-01

**Authors:** Ramón Cuadrado, László Oroszlány, László Szunyogh, Gino Hrkac, Roy W. Chantrell, Thomas A. Ostler

**Affiliations:** 1 Catalan Institute of Nanoscience and Nanotechnology (ICN2), CSIC and BIST, Campus UAB, Bellaterra, 08193 Barcelona Spain; 2 Universitat Autonoma de Barcelona, Cerdanyola del Valles, Bellaterra, 08193 Spain; 3 Department of Physics of Complex Systems, Eötvös University, Pázmány Péter Sétány 1/A, Budapest, H-1117 Hungary; 4 Department of Theoretical Physics, Budapest University of Technology and Economics, Budafoki út 8, H-1111 Budapest, Hungary; 5 MTA-BME Condensed Matter Research Group, Budapest University of Technology and Economics, Budafoki út 8, H-1111 Budapest, Hungary; 6 College of Engineering, Mathematics and Physical Sciences, The University of Exeter, Exeter, EX4 4SB United Kingdom; 7 Institute for Analysis and Scientific Computing, TU Wien, Vienna, Austria; 8 Department of Physics, The University of York, Heslington, York, YO10 5DD UK; 9 Faculty of Arts, Computing, Engineering and Sciences, Sheffield Hallam University, Howard Street, Sheffield, S1 1WB UK; 10 Départment de Physique, Université de Liège, Liège, B-4000 Belgium

## Abstract

The complex magnetic properties of Fe/Ir/Fe sandwiches are studied using a hierarchical multi-scale model. The approach uses first principles calculations and thermodynamic models to reveal the equilibrium spinwave, magnetization and dynamic demagnetisation properties. Finite temperature calculations show a complex spinwave dispersion and an initially counter-intuitive, increasing exchange stiffness with temperature (a key quantity for device applications) due to the effects of frustration at the interface, which then decreases due to magnon softening. Finally, the demagnetisation process in these structures is shown to be much slower at the interface as compared with the bulk, a key insight to interpret ultrafast laser-induced demagnetization processes in layered or interface materials.

## Introduction

The properties of interfaces between different materials that are made to exist side-by-side often differ significantly from the bulk. The aim of creating such interfaces is often to attempt to exploit the properties of one material or another, or to functionalise or tailor specific properties^1^. The possible combinations of e.g. different materials, thicknesses and number of layers is almost infinite and provides huge opportunities for the development of new technologies for a wide range of applications. Such examples include; control of skyrmions and vortex cores for spintronic applications^2–4^; next generation storage devices^5–8^; magnetic tunnel junctions^9–11^; or even low energy electric field control of magnetism in composite multiferroics^12–14^.

Whilst there is huge potential to engineer these types of multilayer structures they are notoriously challenging to control. Control and growth of sharp, clean interfaces requires a painstakingly large amount of work and expertise. Due to lattice mismatch it can be extremely difficult to create “well behaved” interfaces and this inevitably means that strain is present^8,15^ which results in localised modifications of the underlying electronic structure and therefore magnetic properties. Furthermore, many materials that are proposed for technological applications must be in the “ultrathin” regime so that they can be efficiently integrated into devices. This poses a further problem in that the properties that initially existed in the bulk can be completely destroyed in this limit.

A further, rather interesting scientific problem in developing new materials, is how one can understand the microscopic structural, electronic and magnetic degrees of freedom in such interface systems as well as their thermodynamic properties. A number of experimental methods have been developed to characterise atomic structures and interfaces, using for example, x-ray diffraction^15–17^ or electron microscopy^17,18^. Spatial magnetic contrast can often be gained using the magneto-optical kerr effect^19^, magnetic force microscopy^20^ or even using X-ray scattering measurements^21^. Temporal information is also achievable using pump-probe setups^22^ or time-resolved x-ray diffraction^16,23^ (for structural properties). Complex combinations of some of these measurement techniques also allow for time, spatial and even element resolved measurements simultaneously^21,24^, though these tend to require advanced experimental facilities.

The presence of interfaces that are embedded in a sample provides an extra layer of complexity to understand the resulting magnetic properties. In such structures, atoms become inequivalent due to the absence of translational invariance perpendicular to the interface. This means that each plane of atoms experiences different electronic and structural environments. Zakeri and co-workers developed a method for measuring collective magnetic excitations (magnons) using spin-polarised electron energy loss spectroscopy^25^ in an embedded interface of Fe on Ir, allowing access to the underlying magnetic exchange interactions. These kinds of challenging experiments can provide a great deal of information about the magnetic system in a quasi-equilibrium measurement, but achieving time and spatial (depth and in-plane) measurements of magnetic interfaces remains a key challenge.

In the present study, we have combined electronic structure calculations with a thermodynamic model to investigate the detailed electronic, structural and magnetic properties of Fe/Ir/Fe systems. These types of systems have received attention recently due to their potential to be used to engineer skyrmions for spintronics applications^2^. Electronic structure calculations show highly non-trivial exchange interactions which lead to frustration at the interface between Fe and Ir. Our thermodynamic spin dynamics calculations have been used to calculate the quasi-equilibrium and dynamic properties of the magnetic system. By extracting the exchange stiffness – the resistance of magnetisation to twists or noncollinearities – we have demonstrated an unexpected increase with temperature, where usually thermal fluctuations lead to softening of the spinwaves (a decrease in stiffness). The exchange stiffness is an important property to be able to engineer as it hugely affects the dynamics of the spins. As well as affecting the dynamics, it also affects the presence of topological ground state structures, such as vortex cores or skyrmion structures. We conclude the work by investigating the layer-by-layer ultrafast demagnetisation process, which has been widely investigated in the bulk, but recent experiments have shifted towards multilayer structures^5,6,26,27^. Our results reveal that the demagnetisation process is strongly dependent on the environment for the spins and in particular the interface spins demagnetise much more rapidly than the bulk. These observations will be important for understanding ultrafast laser-induced demagnetisation experiments on layered structures.

Electronic structure methods based on density functional theory^28,29^ (DFT) are widely used in condensed matter physics and beyond and can give detailed information of the optimized atomic structure and their electronic and magnetic ground states. However, standard DFT does not generally describe time-resolved information (dynamics), with the exception of its time dependent form^30^ (time-dependent density functional theory, or TDDFT), which is often limited to just a few unit cells due to the high computational cost. Here, we use a multiscale approach involving a combination of *ab-initio* methods and atomistic models with *ab-initio* parameterisation to study the structural, electronic, magnetic and thermodynamic properties of four different *bcc*-Ir thicknesses sandwiched by two semi-infinite Fe regions: … Fe/n_Ir_Ir/Fe … [n_Ir_ = 2, 4, 6, 8], where n_Ir_ represents the number of Ir planes between the Fe slabs.

The methodology applied here is based on three consecutive steps: (1) Structural optimization; (2) Selfconsistent electronic and magnetic parameter calculation, followed by; (3) Spinwave and thermodynamic and spin dynamics calculations. The first step was carried out by means of fully ionic conjugate gradient relaxation at the scalar–relativistic level using the SIESTA based DFT package^31^. In the second step the fully relativistic screened Korringa-Kohn-Rostoker^32^ (SKKR) method is used to determine plane-by-plane anisotropy, magnetic moments (MMs) and magnetic exchange tensor, ${{\mathscr{J}}}_{ij}$. The magnetic anisotropy (MAE) is defined as the energy that it takes to rotate the magnetization direction from the easy into the hard direction of the system. In the present work it will be obtained by means of the magnetic force theorem and further details about the calculation procedure can be found within the Methods section. The magnetic exchange interactions between atoms represent the largest energy term in our magnetic Hamiltonian and is responsible for the type of magnetic ordering (ferromagnetic, antiferromagnetic or exchange spirals). Information on how the exchange magnetic tensors are calculated can be found in the Methods section. This *ab–initio* information is then used in the third step where we carried out spin dynamics simulations based on an extended Heisenberg model to calculate spinwave and thermodynamic properties as well as dynamic properties. Some of the important details of the calculations are presented in the main part of the article, where necessary, with full details outlined in the methods section.

## Electronic Structure Calculations

**Figure 1:**
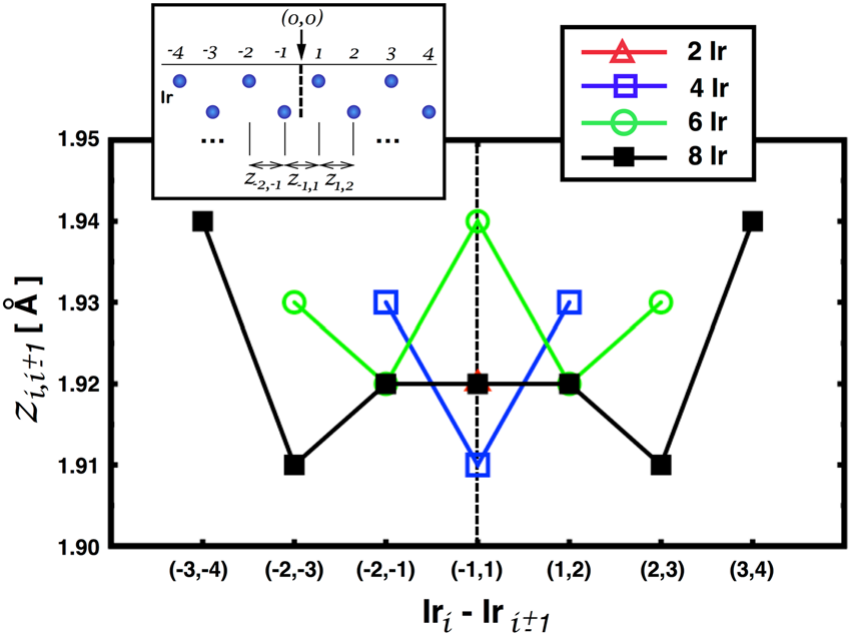
Out–of–plane distances between different Ir planes for … Fe/n_Ir_Ir/Fe … [n_Ir_ = 2, 4, 6, 8] geometries. The center of each configuration has been shifted to the vertical dashed line that represents the central out–of–plane distance for any configuration. The *x*-axis values depict each Ir layer pair, *i* and *i* ± 1.

As described above and in the methods section, we have relaxed the ionic positions of each Ir thickness in Fe/Ir/Fe using the SIESTA code. Figure 1 shows the out–of–plane distances between adjacent Ir planes from the interface to the center of the sandwich. After inspection we observe that the dispersion in the global out–of–plane Ir-Ir distance, after the optimization, is less than 0.03 Å for all the configurations depicting, in general, small deviations. Furthermore, the deviations are more pronounced close to the interfaces, i.e., for the first interfacial Ir layers (n_Ir_ > 2). In addition, it is clear that the out-of–plane distances in the middle stabilize for thicker geometries (black squares), resulting in a disappearance of the *d*_*Ir* − *Ir*_ oscillations seen for n_*Ir*_ < 8.

These relaxed and symmetrized coordinates (see methods) are then passed to the SKKR code. This relaxation step is important to ensure that the magnetic properties are continuous and correctly described. As was shown in Ref.^33^, in the absence of structural relaxation, the domain wall profile becomes much sharper accompanied by a reduced coercivity.

The fully relativistic screened Korringa–Kohn–Rostoker^32^ (SKKR) code was used to calculate the tensorial exchange interactions as well as layer resolved MMs and anisotropies of the relaxed Fe/Ir/Fe structures. Within the SKKR formalism, *K* can be decomposed into site–resolved contributions, *K*_*i*_:

1 $$K=\sum _{i}{K}_{i}$$

**Figure 2:**
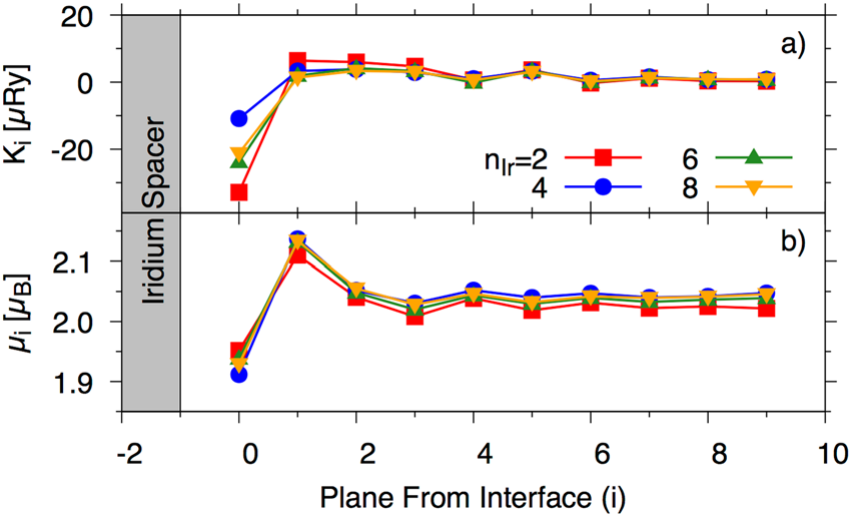
(**a**) Plane–by–plane first order uniaxial anisotropy constant, K_*i*_ and (**b**) magnetic moments, as a function of distance from the Ir interface. The data presented is for n_Ir_ = 2, 4, 6 and 8 planes of Ir (red, blue, green and gold respectively) as shown schematically in Fig. 11 (see methods).

The plane–by–plane anisotropy constants are shown in Fig. 2a), shown as a function of distance from the Ir interface for n_Ir_ = 2, 4, 6 and 8 (red square, blue circle, green upwards triangles and gold downwards triangles respectively). For full details of the method used to calculate the anisotropy see the methods section and references therein.

The anisotropy at the interface is strongly negative (preferentially in–plane) as compared to the bulk value. After the first Fe from the interface, the anisotropy remains positive and shows only a weak variation with distance. The interface anisotropy is almost an order of magnitude larger than in the bulk. The MMs are also shown in Fig. 2b) and display a similarly strong variation with distance from the interface, though the relative change with n_Ir_ is smaller. There is an immediate reduction of around 0.1 *μ*_B_ (compared to bulk) in the atomic magnetic moment within the first Fe atomic plane and an increase in the second followed by small oscillations that quickly become bulk-like from the third plane. The hybridization between Fe and Ir *d* states will promote the rearrangement of the up/down Ir states by means of charge transfer between them leading to the population of iron minority states at the interface, and consequently, a reduction of their MMs. This Fe–*d* hybridization tends to stabilize after the third Fe plane, meaning that the Ir influence on Fe is mainly up to two unit cells towards the Fe bulk.

**Figure 3:**
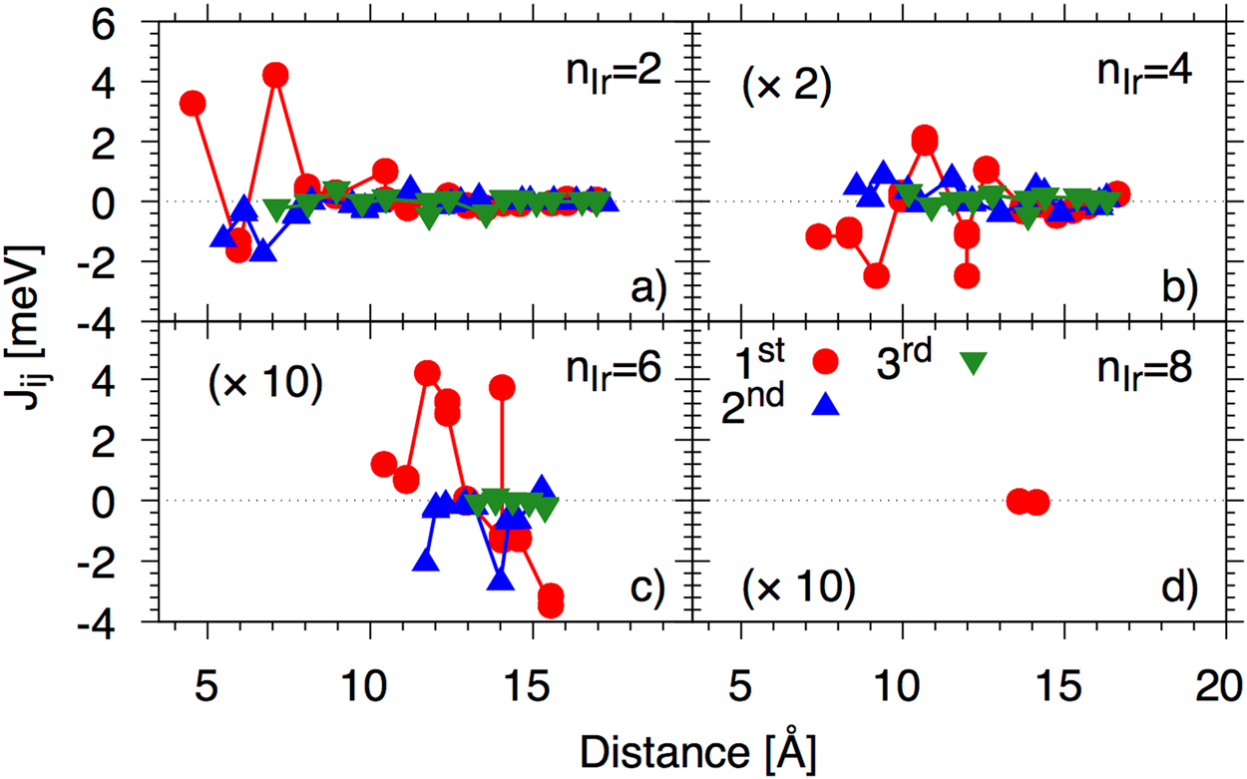
Exchange interactions between an interface Fe spin and the first (red circles), second (blue upwards triangles) and third (green downwards triangles) neighbour planes at the other side of the interface of thickness n_Ir_ = 2, 4, 6 and 8 planes. For the n_Ir_ = 4, 6 and 8 data sets the exchange has been multiplied by 2, 10 and 10 respectively (as shown in the brackets of their respective panels).

The tensorial exchange interactions are calculated within the SKKR code in the spirit of Liechtenstein^34^ (see methods). Due to the lack of translational invariance in the direction perpendicular to the plane, each plane of Fe interacts differently with the other planes (both towards and away from the interface). The focus of the present work is on the effects of the Ir thickness and how the exchange interactions govern the ground state and thermal properties of the system. Figure 3 therefore shows the isotropic part of the exchange interactions between one Fe magnetic moment located at the interface with Ir and the Fe spins at the other side of the interface for each Ir thickness (n_Ir_).

Panels a–d of Fig. 3 show the exchange across the interface of thickness 2, 4, 6 and 8 planes of Ir respectively, with the nearest (1st, red circles), second nearest (2nd, blue upwards triangles) and third nearest (3rd, green downwards triangles) planes. For n_Ir_ = 2, the interaction with the nearest plane is dominated by first and third nearest neighbour ferromagnetic interactions and shows a highly oscillatory behaviour consistent with an RKKY interaction mediated by the conduction electrons. The exchange quickly decays beyond 10 Å and there is a rather small interaction in the third plane. Interaction with the second plane remains negative, though small.

For n_Ir_ = 4, the interaction with the nearest plane is mostly antiferromagnetic, though this also oscillates in a similar way as for n_Ir_ = 2, though the magnitude is much smaller and the total number of neighbours should also be taken into consideration when considering the final ground state spin structure. For n_Ir_ = 6 the value of the exchange is greatly reduced but is dominated by ferromagnetic interactions and for n_Ir_ = 8, the two layers are almost decoupled. It should be noted that in the n_Ir_ = 2, 4 and 6 cases there is a competition between the ferromagnetic and antiferromagnetic contributions at different distances, thus we expect (and indeed observe) the interface to be somewhat frustrated.

**Figure 4:**
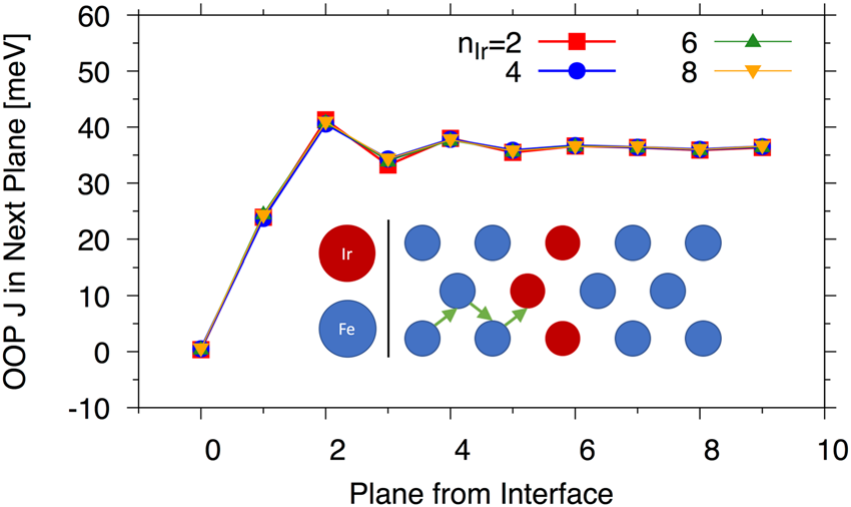
Out-of-plane nearest neighbour exchange acting on Fe planes as a function of plane index from the interface. The exchange value plotted here is the exchange with the next plane towards the interface (as shown schematically in the inset) with the value at the plane index 0 corresponding to the exchange with the first Ir layer.

It should also be noted that the ground state will be determined by, not only the size and sign of the exchange at the interface spin moment with the layers at the opposite side, but by all of the exchange interactions across the interface and the total number of neighbours should also be taken into consideration. However, the data presented in Fig. 3 will be used to interpret the results of the spin dynamics calculations shown in the following section. It is also important to note that in the spin dynamics model (see proceeding section) we do not account for the Ir atoms. This is justified if we consider; (i) the largest Ir moment at the interface (with the largest induced moment) was 0.081 *μ*_B_ for n_Ir_ = 4 (similar values for 6 and 8) and the lowest value was 0.039 *μ*_B_ for n_Ir_ = 2; and (ii) the Fe/Ir exchange was significantly smaller than the bulk (less than 1% taking the nearest out-of-plane distance) as shown on Fig. 4.

Figure 4 shows the nearest neighbour out-of-plane exchange on the Fe sites as a function of distance from the interface. The exchange that is plotted is shown on the inset schematic. As compared to the exchange across the interface (see Fig. 3), for example for n_Ir_ = 6, the Fe-Ir exchange at the interface is around 15–20% that of the Fe-Fe exchange across the interface. The Ir would therefore have a very small effect on the thermodynamic quantities in the spin model. Overall, the Fe-Ir exchange is much smaller than that of the Fe-Fe interactions and this, combined with the very small induced moment of Ir, lead us to neglect the Fe-Ir interactions. Furthermore, the induced moment of the Ir is not trivially described within the atomistic spin dynamics formalism and would require a more advanced theoretical construct.

All of the quantities presented until now are calculated without the presence of thermal fluctuations. In the proceeding section we outline a spin dynamics model based on the Heisenberg Hamiltonian. This thermodynamic model takes, as input, the quantities calculated using *ab-initio* calculations and introduces thermal fluctuations to determine the temperature dependence of the magnetic properties.

## Equilibrium Thermodynamic Properties

**Figure 5:**
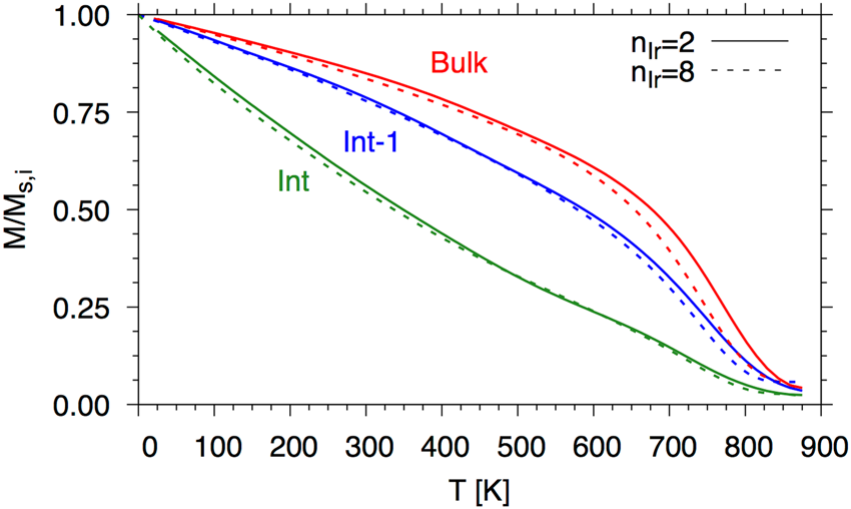
Layer-wise temperature dependent magnetization as a function of temperature. The interface magnetization (Int) has a largely reduced magnetization as compared with the centre of the Iron layer (Bulk). The second plane from the interface (Int-1) recovers its magnetization rather rapidly as the number of missing exchange interactions are reduced.

The equilibrium thermodynamic properties (and the dynamic properties in the proceeding section) make use of a spin model with the energetics based on the calculated Heisenberg Hamiltonian. The time-resolved dynamics are found by solving the Landau-Lifshitz-Gilbert (LLG) equation, which allows one to include on-site parameters, such as, anisotropy (*K*_*i*_), thermal bath coupling (*λ*_*i*_), gyromagnetic ratio (*γ*_*i*_) and exchange (${{\mathscr{J}}}_{ij}$), making the approach ideal to look at finite temperature properties of the Fe/Ir/Fe system. Furthermore, it is possible to determine layer-by-layer thermodynamic properties. We should point out here that we do not simulate the Ir magnetic moments which only appear due to the Weiss field from the Fe, however the effect of the presence of the Ir interface is represented in the Hamiltonian for each of the Fe planes. Full details of the spin dynamics model can be found in the methods section. In Fig. 5 we present numerically determined magnetization curves for two thicknesses of Ir; n_Ir_ = 2 and 8 planes.

**Figure 6:**
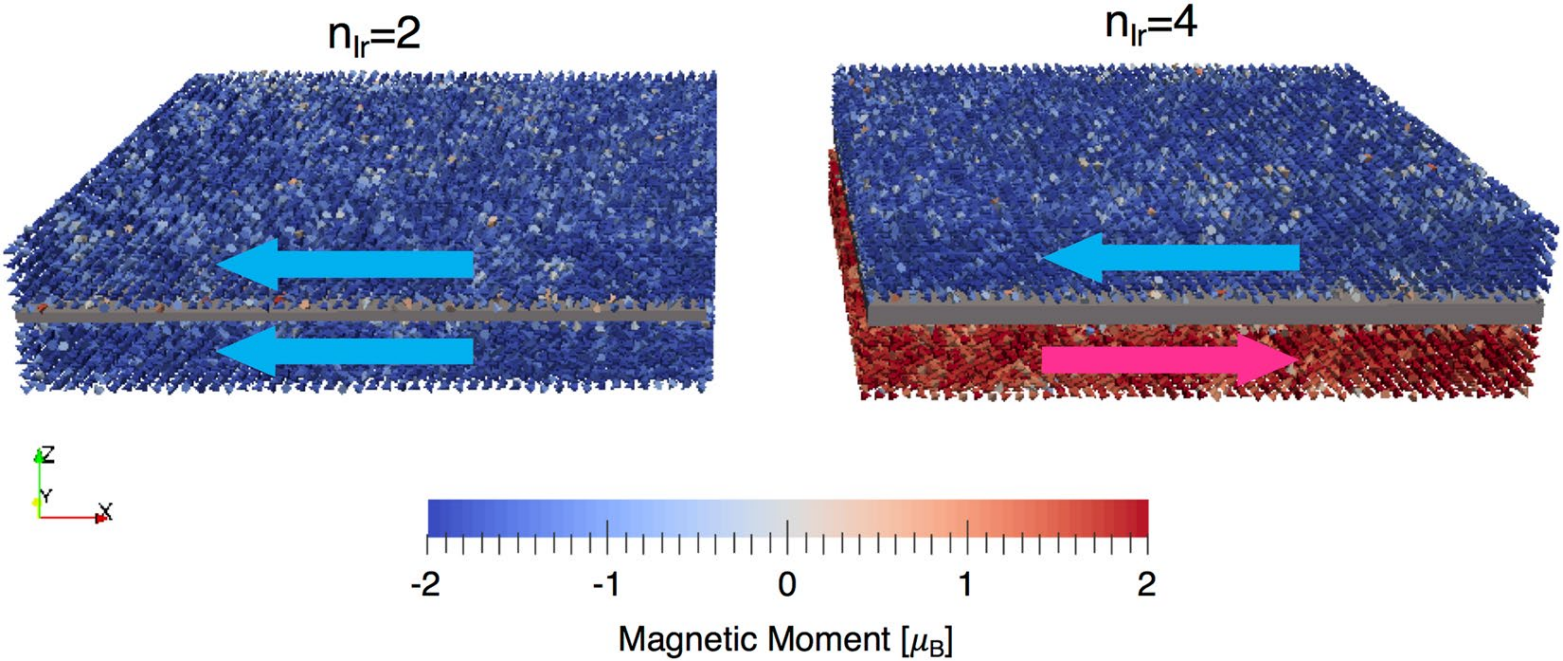
Spin configurations for n_Ir_ = 2 and 4 layers, depicted by the grey cuboid boxes between the Fe spins represented by coloured cones. For n_Ir_ = 2 (left) the two sides of the interface are aligned (FM) and for n_Ir_ = 4 (right) the two sides are overall antiferromagnetically coupled.

The data shown in Fig. 5 is for the two Fe planes nearest to (Int and Int-1) the interface with Ir and one in the centre of the Iron layer (bulk-like). We take the critical damping regime (*λ*_*i*_ = 1.0) which is the spin dynamics analogy of quenched molecular dynamics and equilibrate the magnetization for 20 ps followed by a further (maximum) of 30 ps whereby the average of the magnetization is taken, unless convergence in both the mean and variance of the reduced magnetization of each plane reached 10^−6^ and 10^−7^ respectively. For n_Ir_ = 2, 4, 6 and 8 the magnetization curves are similar with similar phase transition (Curie) temperatures. The interface layer shows a largely reduced magnetization over the bulk planes due mostly to “loss” of exchange because of the presence of Ir. In all four cases the phase transition temperature is dominated, as expected, by the bulk exchange interactions, though there is a slight reduction in the magnetization (difference between dashed and solid lines in Fig. 5 of the same colour) for a given plane. The exchange does, however, modify the ground state. For each value of n_Ir_ we have calculated a ferromagnetic ($\leftleftarrows$) and an antiferromagnetic ($\leftrightarrows$) alignment to check for the stability as a function of temperature (see example spin configurations in Fig. 6).

As shown for n_Ir_ = 2 and 4 in Fig. 6, modifying the size of the Ir spacer modifies the effective exchange interaction across the interface. For n_Ir_ = 2 and 4 the ground state was found to be FM and AFM respectively, whereas for n_Ir_ = 6 the case was again FM. For n_Ir_ = 8 (beyond 2 nm) the separation between the Fe atoms at either side of the interface was so large that the two sides of the interface were essentially exchange decoupled, though the long-range dipole-dipole interaction would still lead to some coupling. Whilst we predicted from Fig. 2 that the ground state for n_Ir_ = 2 and 4 would be FM and AFM respectively, the competition of exchange interactions leads to some frustration and a reduced magnetization as we saw in Fig. 5, thus it is not trivial to predict the ground state as it is the result of the exchange interactions at either side of the interface (including taking into account the total number of neighbours at a given distance). Table 1 shows the Curie temperature dependence on the Ir thickness as well as the ground state configuration, which can be either ferromagnetic (FM), antiferromagnetic (AFM) or decoupled (DC).

**Table 1:** Curie temperature dependence and ground state configuration as a function of the Ir thickness. FM means ferromagnetic, AFM is antiferromagnetic and DC means decoupled. The errors in the estimated Curie temperatures stem from the fitting procedure described in the methods section.

n_Ir_	T_C_	Ground State
2	784.62 ± 1.62	FM
4	780.61 ± 2.19	AFM
6	786.53 ± 5.63	AFM
8	777.12 ± 1.61	DC

Table 1 shows that, as a function of n_Ir_, the Curie temperature does not vary much within the fitting error. However, by varying the thickness, the ground state configuration changes from FM to AFM and then to a decoupled state due to the low exchange in the n_Ir_ = 8 case.

**Figure 7:**
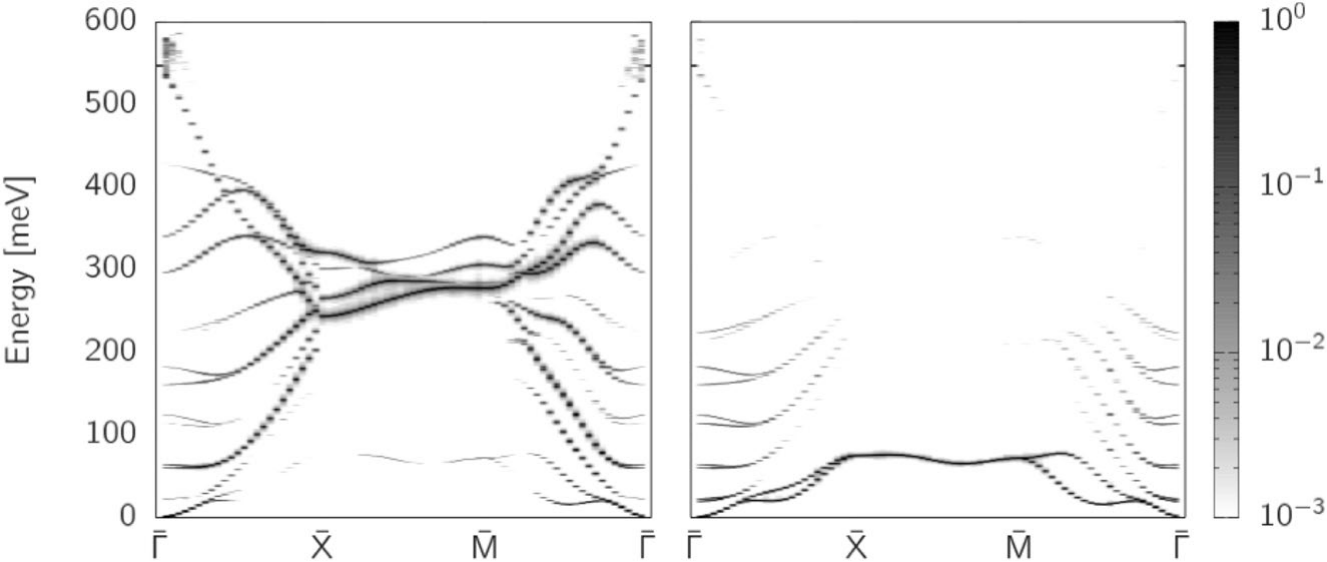
Layer resolved spinwave dispersion (in-plane) along the pseudo-cubic path $\bar{{\rm{\Gamma }}}\to \bar{X}\to \bar{M}\to \bar{{\rm{\Gamma }}}$. Left is for a bulk layer and the right is for the Fe at the interface with Ir. The lowest energy mode is much more localised in the interface Fe.

In Ref.^25^ the spinwave modes in an Fe/Ir/substrate system were measured experimentally (acoustic branch only) and theoretically. Theoretical modeling predicted that the modes at the interface are localized at this interface, which manifests as a higher spinwave intensity in the spectral function of the transverse susceptibility for the interface Fe. In Fig. 7 we show the spinwave modes for Fe bulk (left) and for Fe at the Ir interface in the case of n_Ir_ = 2 (right). The path is presented in the 2D Brillouin zone and is calculated for 10 planes of Fe at either side of the interface, though we present here just two of those planes. For an interface, one expects as many branches as there are atoms, however, as the atoms at either side of the interface are equivalent there is a degeneracy for each branch except at $\bar{{\rm{\Gamma }}}$ where a splitting is seen.

The modes for n_Ir_ show a similar localisation of the lowest energy mode at the interface close to $\bar{{\rm{\Gamma }}}$. Furthermore, the higher energy optical modes are less localised for the interface layers (right) as demonstrated by the lack of contrast, consistent with Ref.^25^. For the curves in Fig. 7 we normalise the amplitude at each *k*-point so that the branch with the highest amplitude has a value of 1 allowing us to compare the relative amplitudes for a given *k*.

The spinwave dispersion for the other Ir thicknesses are quite similar (not shown) in their structure. However, for the other thicker Ir sandwich structures the splitting that occurs towards the $\bar{{\rm{\Gamma }}}$ point does not occur (or we do not have the resolution to resolve it) due to the rather weak coupling across the interface, meaning that they almost act as individual interfaces (rather than sandwiches) in terms of the spinwave properties in the plane. Whilst these low temperature calculations reveal the complex behaviour of the spinwave dispersion, the more experimentally accessible quantity is the exchange stiffness, *A*, which is usually found by fitting the spinwave dispersion to *ω* = *Ak*^2^ at low-k, requiring, for example, neutron scattering measurements. The stiffness can also be found using Bloch’s law from the equilibrium magnetization curve^35^ or using ferromagnetic resonance experiments^35^, though both of these measure very long wavelength effects close to Γ. Due to increasing spin disorder due to thermal fluctuations, the exchange stiffness generally decreases with temperature (it becomes easier to induce twists in the magnetic structure).

**Figure 8:**
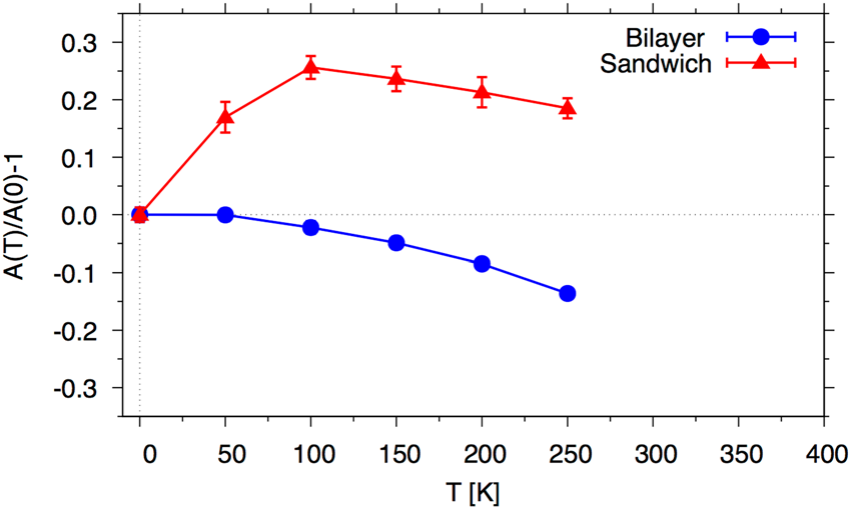
Change in in-plane exchange stiffness as a function of temperature for n_Ir_ = 2 as measured from the spinwave dispersion. The upper (red) curve shows that there is initially an increase in the stiffness which we attribute to the frustration at the interface which decreases with temperature. By artificially removing one side of the interface there is a consistent decrease in the stiffness (lower blue curve).

We have analysed the spinwave data from the spin dynamics calculations by fitting the lowest branch to the function, *ω* = *Ak*^2^ up to *ka* = 1.2 (note that this is the in-plane stiffness constant only). In Fig. 8 we show the change in the stiffness compared to the value at 0 K (i.e. the relative change), *A*(*T*)/*A*(0) − 1, as a function of temperature.

The data in Fig. 8 is presented for n_Ir_ = 2 and interestingly, as a function of temperature, there is an initial increase in the exchange stiffness (red triangle points in Fig. 8). This increase, we believe, arises from the frustration at the interface which decreases with increasing thermal energy and leads to an increased relative alignment in the planes. This increased alignment means that it is more difficult to induce long-range variations in the magnetic order resulting in an increased in-plane stiffness. After around 100 K the thermal fluctuations become dominant and the normal decrease in the stiffness is seen. To verify that the presence of the interface is resulting in an increased stiffness we performed the same calculations but only including one side of our sandwich to make an Fe/Ir bi-layer. We note that we did not repeat the electronic structure calculations meaning that it is more of a computational thought experiment. Indeed, in this case we observe an increase in the stiffness for all temperatures (blue circle points in Fig. 8) demonstrating the importance of the interface.

## Dynamic Properties

So far we have presented purely static/equilibrium first principles and thermodynamic results. In the final part of this article we present layer resolved dynamics of our interface system after excitation with short laser pulses. It has been previously shown^36^ that the time-scale of the dynamics of magnetic materials after short laser pulses are governed primarily by a combination of the magnetic moment and the exchange interaction, $\tau \sim \mu /J$. In Ref.^36^, a systematic study of a range of samples of different magnetic ordering (ferromagnetic and ferrimagnetic), and alloyed with different elements showed a linear scaling of the demagnetization time with magnetic moment. As we saw in Fig. 2, there is a reduction of the Fe magnetic moment directly at the interface and a slight increase in the next layer before becoming *bulk*-like. The variation in the magnetic moment is rather small but a reduction in the moment should see a slight decrease in the relaxation time. On the other hand, there is a reduction in the exchange interaction at the interface which would see an increase in the relaxation time. Here we focus on the n_Ir_ = 2 system. We assume that the value of the coupling constant, *λ*_*i*_, is the same for each plane so as to compare the effects of the exchange and the magnetic moment.

**Figure 9:**
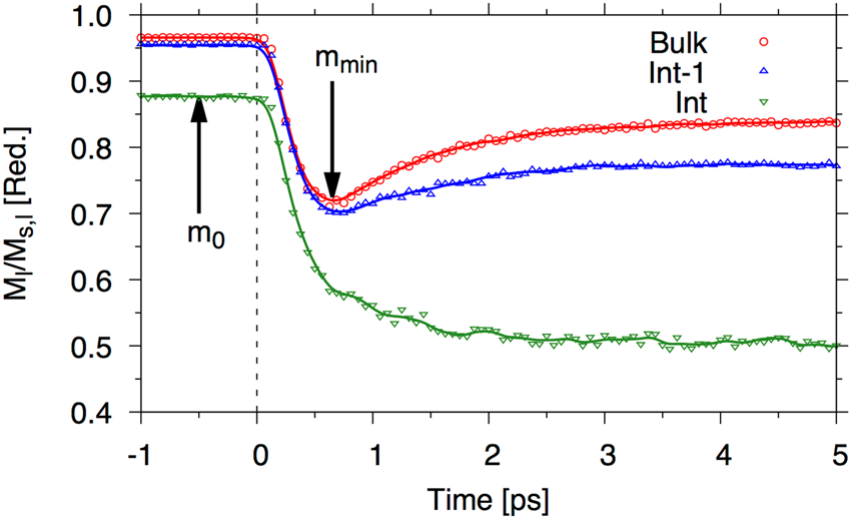
Example magnetization dynamics data for a range of layers. The points represent the data taken from the spin dynamics model and the lines are Gaussian smoothed data with a width of 75 fs.

To model the heating effect we take a simple two-temperature model which allows us to define an electron and a phonon temperature which is required for the correlator (see methods). Transient changes in the electronic temperature due to a laser pulse give rise to increasing thermal fluctuations in the spin system. Our focus here is on the difference between the relaxation times at different sites from the interface. Figure 9 shows an example of the magnetization dynamics for the bulk, interface (Int) and plane next to the interface (Int-1) for a moderate pump fluence corresponding to a temperature increase of around 250 K.

As we see from Fig. 9 the dynamics of the different layers are quite different. In the bulk, the magnetization initially demagnetises and then begins to recover. For the interface layer the magnetization shows the same initial rapid drop but then continues to decrease at a slower rate and never recovers. To determine the relaxation time we normalise the data for each layer (*l*) by ${m}_{n}^{l}(t)=\frac{{m}^{l}(t)-{m}_{min}^{l}}{{m}_{0}^{l}-{m}_{min}^{l}}$, so that the initial ${m}_{n}^{l}$ value is 1 and decreases to zero at the point where the magnetization is at a minimum. We then fit a single exponential to the data and extract the characteristic demagnetization time associated with the initial part of the demagnetization. This method allows us to consistently define a demagnetization time independently of what the form of the magnetization curve is.

**Figure 10:**
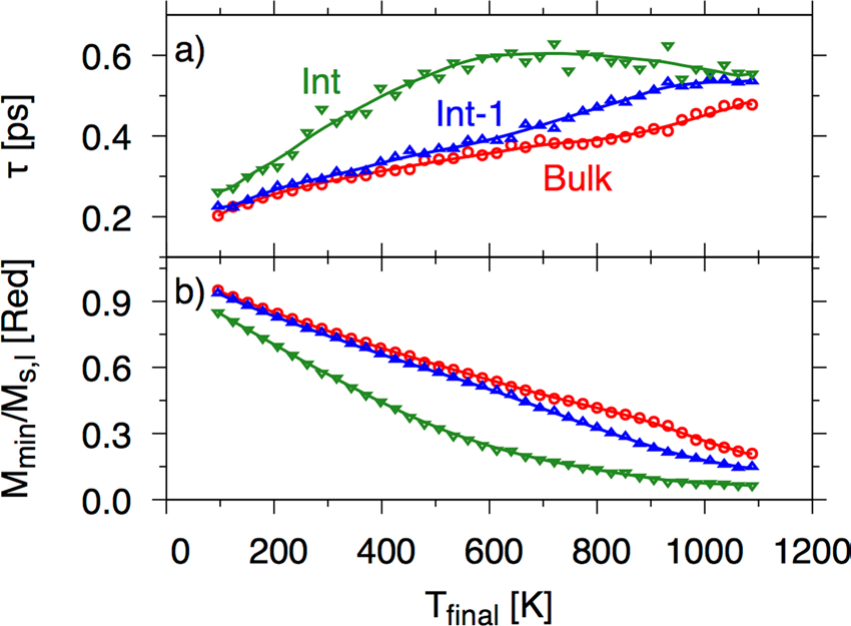
Demagetisation times (**a**) and minimum magnetisation (**b**) as a function of temperature after a 50 fs laser pulse for atoms at (Int) and one plane away from (Int-1) the Ir interface and bulk planes.

In Fig. 10 we present demagnetization rates and the minimum magnetization (note ${m}_{min}^{l}={M}_{min}^{l}/{M}_{s,l}$, where *M*_*s*,*l*_ is the saturation magnetization of that layer, *l)* after the action of a 50 fs heat pulse starting at 82 K, as a function of the final temperature after the laser, T_final_.

As we see from Fig. 10a) the interface demagnetises at a much slower rate than the other planes. Interestingly, the relaxation time actually reaches a maximum value at around T_final_ = 700 K. What is clear from the demagnetisation times is that the exchange contribution is much larger than that of the magnetic moment. The minimum in the magnetization is taken up to a maximum of 25 ps after the pulse. For the interface plane, as expected, is much lower than that of the bulk values (see panel b). For high temperatures (pump fluence) the magnetisation does not quite reach zero after 25 ps due to critical slowing down.

## Discussion

We have applied a hierarchical multiscale procedure to model the electronic, structural and magnetic properties of Fe/Ir/Fe sandwiches. SKKR calculations revealed the plane-by-plane magnet moments, anisotropies and exchange interactions that govern the ground state magnetic configuration. Spin dynamics simulations show that the ground state changes from FM to AFM and finally a decoupled state as the thickness of Ir is increased with a frustration effect present at the interface, which manifests as a reduced magnetisation in the interface Fe planes (and also due to the reduced exchange interaction due to the presence of the interface). The spinwave dispersion is in good agreement with previous measurements. Our theoretical calculations of an embedded interface, however show a splitting of the frequencies approaching the $\bar{{\rm{\Gamma }}}$ point for the strongly coupled n_Ir_ = 2 case, disappearing as the Ir spacer increases in thickness.

Our temperature-dependent spinwave calculations show that the presence of the interface can lead to an initially counter-intuitive increase in the in-plane spinwave stiffness with temperature. Generally, in bulk materials, the increasing thermal fluctuations leads to a reduction in the spinwave stiffness as there is an effective reduction in the exchange due to thermal fluctuations meaning that the system can “twist” more readily (become more non-collinear). For the interface system presented here, we ascribe the initial increase with temperature to a decrease in frustration which leads to a more collinear state. This could be used to engineer the stiffness of systems for magnonics applications or skyrmion based spintronics.

Finally, we investigated the laser-induced demagnetisation process on a plane-by-plane basis. The demagnetization times, which are not measurable experimentally, show a strong difference at the interface, compared with the bulk, and show that the exchange dominates the dynamics in this region. The variation in magnetic moment as a function of distance from the interface is rather small and does not play a large role in the variation in demagnetisation times. In this type of spin model, the thermal bath coupling can also affect the demagnetisation times. This coupling is a phenomenological parameter that attempts to describe the transfer of angular momentum from the magnetic moments to the electronic and phononic degrees of freedom, and could be rather different for each plane. Whilst there are experimental measurements available to measure time, element and spatially resolved dynamics, the resolution is often limited and obtaining information on a plane-by-plane basis with this level of detail is not yet available. Therefore our results will be useful to interpret and understand the fine details of experiments coming from ultrafast demagnetisation experiments on layered structures.

## Methods

### Structural Relaxation with SIESTA

Structural relaxation of each of the Fe/Ir/Fe systems was carried out by means of fully ionic conjugate gradient relaxation at the scalar–relativistic level using the SIESTA DFT package^31^. We used the local spin density approximation (LSDA) as the exchange correlation potential based on Ceperley and Alder’s parametrization^37^. As a basis set, we have employed double-*ζ* polarized (DZP) strictly localized numerical atomic orbitals.

**Figure 11:**
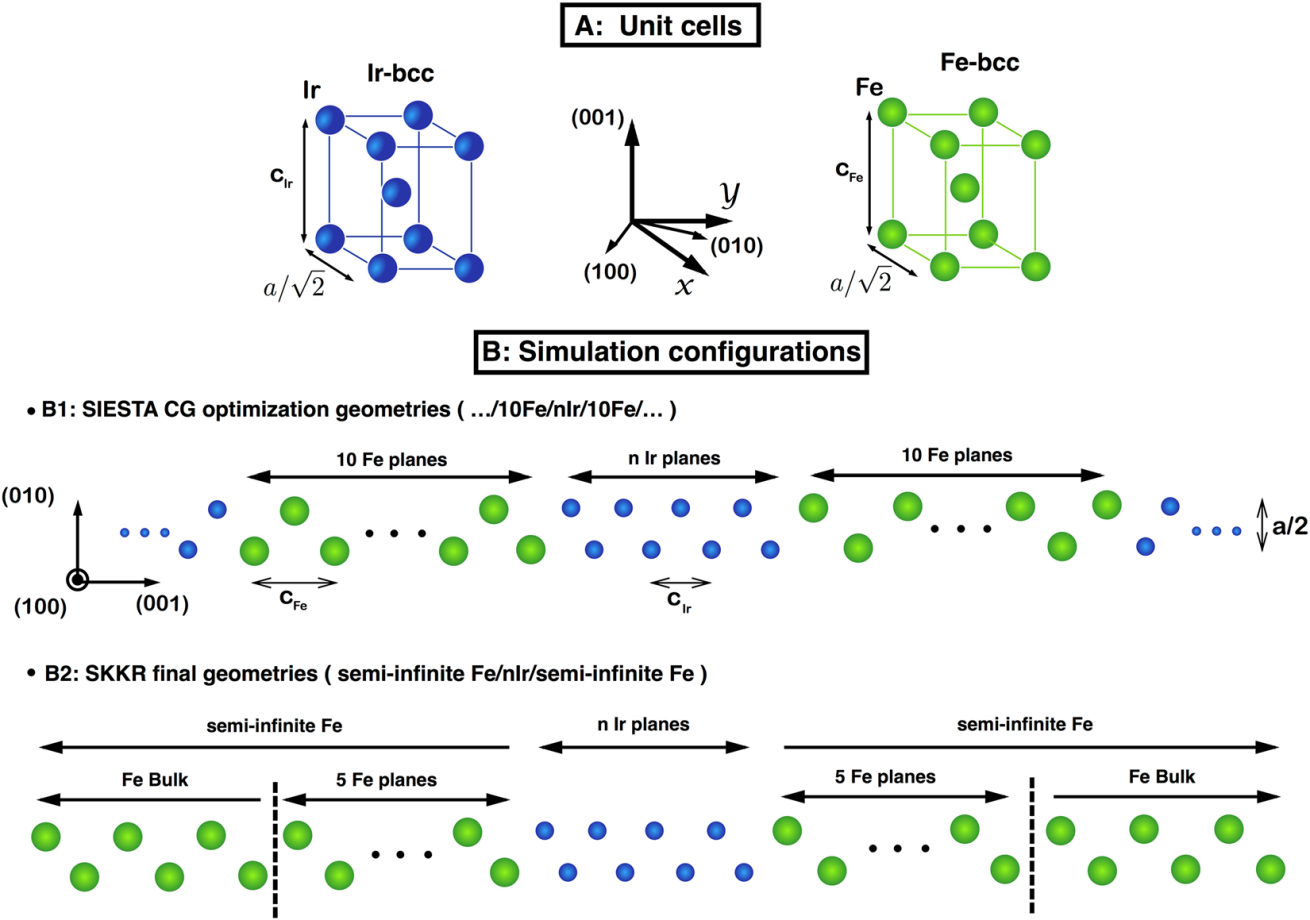
(**A**) Fe and Ir *bcc* unit cells. (**B1**) Schematic side view of the initial atomic structure used in the optimization process (see text for details). (**B2**) Depicts the structure employed in the SKKR calculations after the relaxation in B1. It is composed of two semi-infinite Fe–*bcc* slabs on either side of the Ir slab.

We begin by considering the starting structural ordering of our Fe/n_Ir_ Ir/Fe sandwich. The Fe and Ir structures start with a *bcc* structure^38^ (as shown in the upper panel of Fig. 11). Due to the in–plane mismatch between Ir and Fe bulk lattices we have used a common in–plane lattice constant, *a*, of 3.8467 Å. After the relaxation, a careful inspection of the final atomic *x* and *y* coordinates reveals that the in–plane deviations with respect to the initial positions varies by less than ±10^−3^ Å, because of this, the Ir atoms at different *p* planes will tend mainly to change their out–of–plane distances. To obtain the final … Fe/n_Ir_Ir/Fe … configurations used in the SKKR calculations we carry out the optimization process for each Ir thickness as shown schematically in Fig. 11–B1 for 8 Ir planes. Each supercell contains 20 Fe planes plus n_Ir_ plus 20 Fe planes and repeated periodically along Z coordinate. After the relaxation, the forces per atom were less than 0.02 eV/Å and the energy tolerance on each self–consistent cycle was less than 10^−4^ eV. The mesh cut-off to calculate the real space integrals was 700 Ry and 12 × 12 × 10 k-points were used. It is worth mentioning that during the optimization process, the atomic out–of–plane Ir distances close to the interface had tiny asymmetries of at most ±0.005 Å. This could be due to the uneven character of our configurations that are are not completely symmetric in the simulation unit cell since as is shown in Fig. 11–B1 where perfect mirror symmetry with respect to the *xy* plane (center of the sandwich) is missing. In order to avoid these small deviations in any of the calculated magnetic properties we decided to mirror the left out–of–plane distances with respect to the center of the Ir slice, the results of which are shown in Fig. 1.

### Calculations of Magnetic Properties with SKKR

The fully relativistic screened Korringa–Kohn–Rostoker^32^ (SKKR) code was used to calculate the tensorial exchange interactions as well as layer resolved MMs and anisotropies of the relaxed Fe/n_Ir_ Ir/Fe structures. The LSDA exchange and correlation potential^37^ was employed in the SKKR calculations making use of the atomic sphere approximation^39^. As a first step, self-consistent calculations were performed to converge the potentials which were performed in the fully relativistic mode, solving the full Dirac equation with angular momentum cutoff *l*_max_ = 2, which incorporates the d-electrons and we find that this gives good convergence of the exchange parameters (diagonal and off-diagonal) for Fe based systems, though for other properties (e.g. transport properties) this may not be the case. A semicircular contour with 16 energy points was used for the energy integration and 1680 k-points were used for the self-consistent cycles with a progressive increase above this value used for the calculation of the exchange. The self–consistent calculations were converged to within an energy tolerance of 10^−8^ Ry. We note that this set of parameters corresponds to well converged magnetic model parameters.

In order to investigate the magnetic properties of the Fe/n_Ir_Ir/Fe system at finite temperatures, we use a mapping of the energy of the itinerant electron system onto a classical spin Hamiltonian of Heisenberg form truncated to bilinear term. Writing the spin-spin interactions up to bilinear terms, the Hamiltonian can be written:

2 $$ {\mathcal H} =-\sum _{i}K({{\bf{S}}}_{i})-\frac{1}{2}\sum _{ij}{{\bf{S}}}_{i}{{\mathscr{J}}}_{ij}{{\bf{S}}}_{j}-\sum _{i}{{\mu }}_{i}\cdot {\bf{B}}$$

where the first term is the on-site anisotropy energy and the second term is the exchange, with ${{\mathscr{J}}}_{ij}$ being 3 × 3 matrices, and the final term is the Zeeman energy due to the applied magnetic field, **B**.

For the calculation of the magnetic anisotropy energy we used the magnetic force theorem, where the total energy of the system can be replaced by the single–particle (band) energy. Employing the torque method^40^, in leading (second) order of the spin-orbit coupling, the uniaxial magneto-crystalline anisotropy constant, K, can be calculated as (see Ref.^33^):

3 $$K=E(\theta ={90}^{\circ })-E(\theta ={0}^{\circ })=\frac{dE}{d\theta }{|}_{\theta ={45}^{\circ }}$$

where *θ* is the angle of the magnetization direction with respect to the [001] direction (i.e. perpendicular to the interface). Within the SKKR formalism, *K* can be decomposed into site–resolved contributions, *K*_*i*_:

4 $$K=\sum _{i}{K}_{i}$$

Full details on the torque method can be found in Ref.^41^. The plane–by–plane anisotropy constants are shown in Fig. 2a), shown as a function of distance from the Ir interface for n_Ir_ = 2, 4, 6 and 8 (red square, blue circle, green upwards triangles and gold downwards triangles respectively).

The ${{\mathscr{J}}}_{ij}$ matrices are less straightforward to calculate. The exchange tensor can be divided into three terms^42^:

5 $${{\bf{S}}}_{i}{{\mathscr{J}}}_{ij}{{\bf{S}}}_{j}={J}_{ij}{{\bf{S}}}_{i}\cdot {{\bf{S}}}_{j}+{{\bf{S}}}_{i}{{\mathscr{J}}}_{ij}^{S}{{\bf{S}}}_{j}+{{\bf{D}}}_{ij}\cdot ({{\bf{S}}}_{i}\times {{\bf{S}}}_{j}\mathrm{).}$$

Here the first and second terms on the right hand side are the isotropic and symmetric anisotropic exchange interactions, respectively and the third term is the Dzyaloshinsky-Moriya (DM) interaction^43,44^, where the **D**_*ij*_ are defined as:

6 $${D}_{ij}^{x}=\frac{1}{2}({J}_{ij}^{yz}-{J}_{ij}^{zy}),\quad {D}_{ij}^{y}=\frac{1}{2}({J}_{ij}^{zx}-{J}_{ij}^{xz}),\quad {D}_{ij}^{z}=\frac{1}{2}({J}_{ij}^{xy}-{J}_{ij}^{yx})$$

The components of the exchange tensor, ${{\mathscr{J}}}_{ij}$ can be conveniently found by taking second derivatives of the Hamiltonian with respect to polar and azimuthal angles of the spins:

7 $$\frac{{\partial }^{2} {\mathcal H} }{\partial {\alpha }_{i}\partial {\beta }_{k}}={\delta }_{ik}{K}^{\alpha \beta }({{\bf{S}}}_{i})+{\delta }_{ik}\sum _{j(\ne i)}{{\bf{S}}}_{i}^{\alpha \beta }{{\mathscr{J}}}_{ij}{{\bf{S}}}_{j}+\mathrm{(1}-{\delta }_{ik}){{\bf{S}}}_{i}^{\alpha }{{\mathscr{J}}}_{ik}{{\bf{S}}}_{k}^{\beta }$$

where *α* or *β* can be either *θ* or *ϕ* (polar and azimuthal angles respectively) and

8 $${K}^{\alpha \beta }({{\bf{S}}}_{i})=\frac{{\partial }^{2}K({{\bf{S}}}_{i})}{\partial {\alpha }_{i}\partial {\beta }_{i}},\quad {{\bf{S}}}_{i}^{\alpha }=\frac{\partial {{\bf{S}}}_{i}}{\partial {\alpha }_{i}},\quad {{\bf{S}}}_{i}^{\alpha \beta }=\frac{{\partial }^{2}{{\bf{S}}}_{i}}{\partial {\alpha }_{i}\partial {\beta }_{i}}$$

As was shown in Ref.^42^, the full exchange matrix and anisotropy contributions can be obtained by taking combinations of the derivatives of the Hamiltonian with respect to the angles and constraining the magnetization along different reference orientations, which are calculated (in the spirit of Lichtenstein^34^) through integration over the scattering path operators to the Fermi energy (see Ref.^42^ for full details). The full exchange tensor was calculated up to a maximum number of 4.5 times the in-plane lattice constant for all the *n*_*Ir*_ thicknesses.

### Atomistic Spin Dynamics

For the spin dynamics we use a model based on the LLG equation for each spin, *i*, which can be written as^45^:

9 $${\dot{{\bf{S}}}}_{i}=-\frac{{\gamma }_{i}}{\mathrm{(1}+{\lambda }_{i}^{2}){\mu }_{s,i}}{{\bf{S}}}_{i}\times [{{\bf{H}}}_{eff,i}+{\lambda }_{i}{{\bf{S}}}_{i}\times {{\bf{H}}}_{eff,i}]$$

where *γ*_*i*_, *λ*_*i*_ and *μ*_*s*,*i*_ are the gyromagnetic ratio, thermal bath coupling constant and the magnetic moment (at site *i*) respectively. **H**_*eff*,*i*_ is the effective field at site *i*, where:

10 $${{\bf{H}}}_{eff,i}=-\frac{\partial { {\mathcal H} }_{i}}{\partial {{\bf{S}}}_{i}}+{\boldsymbol{\zeta }}$$

***ζ*** is a fluctuating stochastic white noise term. The stochastic integrals are interpreted in the Stratonovich form^46^ which has been applied in a number of works^47–49^. By imposing a stationary solution of the Fokker-Planck equation where the time derivative of the probability distribution goes to zero and imposing the Boltzmann distribution it is possible to show that the mean and variance of the stochastic term can be given by equation 11. For more details on the derivation of the correlator see Refs^50,51^.

11 $$\langle {\zeta }_{i,a}(t)\rangle =\mathrm{0,}\quad \langle {\zeta }_{i,a}(t){\zeta }_{j,\beta }(t^{\prime} )\rangle =\frac{2{\lambda }_{i}{k}_{{\rm{B}}}T{\mu }_{i}}{{\gamma }_{i}}{\delta }_{ij}{\delta }_{\alpha ,\beta }\delta (t-t^{\prime} )$$

The LLG equation allows one to include on-site parameters, such as, anisotropy (*K*_*i*_), thermal bath coupling (*λ*_*i*_), gyromagnetic ratio (*γ*_*i*_) and exchange (${{\mathscr{J}}}_{ij}$), making the approach ideal for investigating finite temperature properties of the Fe/Ir/Fe system. Furthermore, it is possible to determine layer-by-layer thermodynamic properties. We should point out here that we do not simulate the Ir magnetic moments which only appear due to the Weiss field from the Fe (see discussion in main text). Our simulations use the whole exchange tensor (more than 1000 neighbours per spin). In systems with translational invariance the exchange field can be conveniently calculated using *fast Fourier transforms* and taking advantage of the convolution theorem. However, here due to the lack of this translational invariance (at least for out of plane interactions), no such method can be applied. We use graphical processing units (GPUs) to accelerate the calculations, however, they still remain computationally rather expensive. We simulate 48 × 48 × 1 repetitions of our SKKR supercell (with 10 Fe planes at either side of the interface). We use the Heun numerical scheme to integrate the LLG equation of motion and use a time-step of 0.1 fs to ensure numerical stability.

### Fitting Procedure to Determine the Curie Temperature

To calculate the Curie temperatures we used a fitting procedure, fitting to the equilibrium magnetization of the bulk planes to the expansion^52^:

12 $$M(T)={a}_{0}\sqrt{\frac{{T}_{{\rm{C}}}-T}{{T}_{{\rm{C}}}}}+\sum _{p=1}^{8}{a}_{p}{(\frac{{T}_{{\rm{C}}}-T}{{T}_{{\rm{C}}}})}^{p}$$

### Spinwave Calculatons

The spinwave dispersion, using the spin dynamics code, can be calculated by calculating the dynamic structure factor^53^:

13 $$\mathscr{S}({\bf{k}},\omega )=\frac{1}{N\sqrt{2\pi }}\sum _{{\bf{r}},{\bf{r}}{\rm{^{\prime} }}}{e}^{i{\bf{k}}\cdot ({\bf{r}}-{\bf{r}}{\rm{^{\prime} }})}{\int }_{{\rm{\infty }}}^{+{\rm{\infty }}}{e}^{-i\omega t}C({\bf{r}}-{\bf{r}}{\rm{^{\prime} }},t)dt$$

where *C*(**r** − **r**′, *t*) = 〈*S*^+^(**r**, 0)*S*^−^(**r**′, *t*)〉 is the spin-spin correlation function of the transverse spin values (*S*_*y*_ and *S*_*z*_ in this case). The stochastic thermal term allows the spin system to sample all modes and the resulting spectra are analyzed to determine the frequencies. To calculate Eq. 13, a low value of the coupling to the thermal bath was used (*λ*_*i*_ = 0.001) giving narrow line -widths for the spinwave eigenfrequencies. A high damping regime was initially used to relax the magnetization to equilibrium followed by 200 ps calculations of the time integral in Eq. 13.

### Two-Temperature Model of Laser Heating

To model the heating effect we take a simple two-temperature model which allows us to define an electron and a phonon temperature through the coupled equation:

14 $$\begin{array}{c}{C}_{e}\frac{d{T}_{e}}{dt}=-G({T}_{e}-{T}_{p})+P(t)\\ {C}_{p}\frac{d{T}_{p}}{dt}=G({T}_{e}-{T}_{p})-{C}_{p}\frac{{T}_{p}-{T}_{eq}}{{\tau }_{p}},\end{array}$$

where *C*_*e*_ and *C*_*p*_ are the electron and phonon specific heats and *C*_*e*_ = *γ*_*e*_*T*_*e*_. *P*(*t*) is a laser source which we assume to be a Gaussian with a width of 50 fs. For the electron phonon coupling factor, *G*, we use a value of 10 × 10^17^ W/m^3^ K^54,55^. *γ*_*e*_ takes a value of 225 Jm^3^ K^−1 ^^55^ and a constant lattice specific heat, *C*_*p*_ = 3.1 × 10^6^. We add an extra term that removes heat from the phonon system at a rate of *τ*_*p*_(=1 ns) which would bring the temperature back to equilibrium, T_*eq*_, on a longer time-scale. Our focus here is on the difference between the relaxation times at different sites from the interface, rather than an accurate description of the two-temperature model^56^.
